# An asymptomatic case of peritoneal encapsulation: case report and review of the literature

**DOI:** 10.1186/1757-1626-3-13

**Published:** 2010-01-09

**Authors:** Omer S Al-Taan, Martyn D Evans, Javid A Shami

**Affiliations:** 1 General Surgery Department, Princess of Wales Hospital, (Coity Road), Bridgend, (CF31 1PU), UK

## Abstract

Peritoneal encapsulation is a rare congenital anomaly characterised by a thin membrane of peritoneum encasing the small bowel to form an accessory peritoneal sac. We present a case of peritoneal encapsulation diagnosed incidentally in an 82 year old man undergoing laparotomy for colonic cancer. The sac was easily excised and surgery was otherwise uneventful. A discussion of the case and a review of the literature are presented.

## Introduction

Peritoneal encapsulation (PE) is a very rare condition that is characterised by a thin membrane encasing the small bowel forming an accessory peritoneal sac. Cases usually present with small bowel obstruction or can be an incidental finding during laparotomy. We present a case of PE diagnosed during surgery for a colon cancer.

## Case report

**Figure 1:**
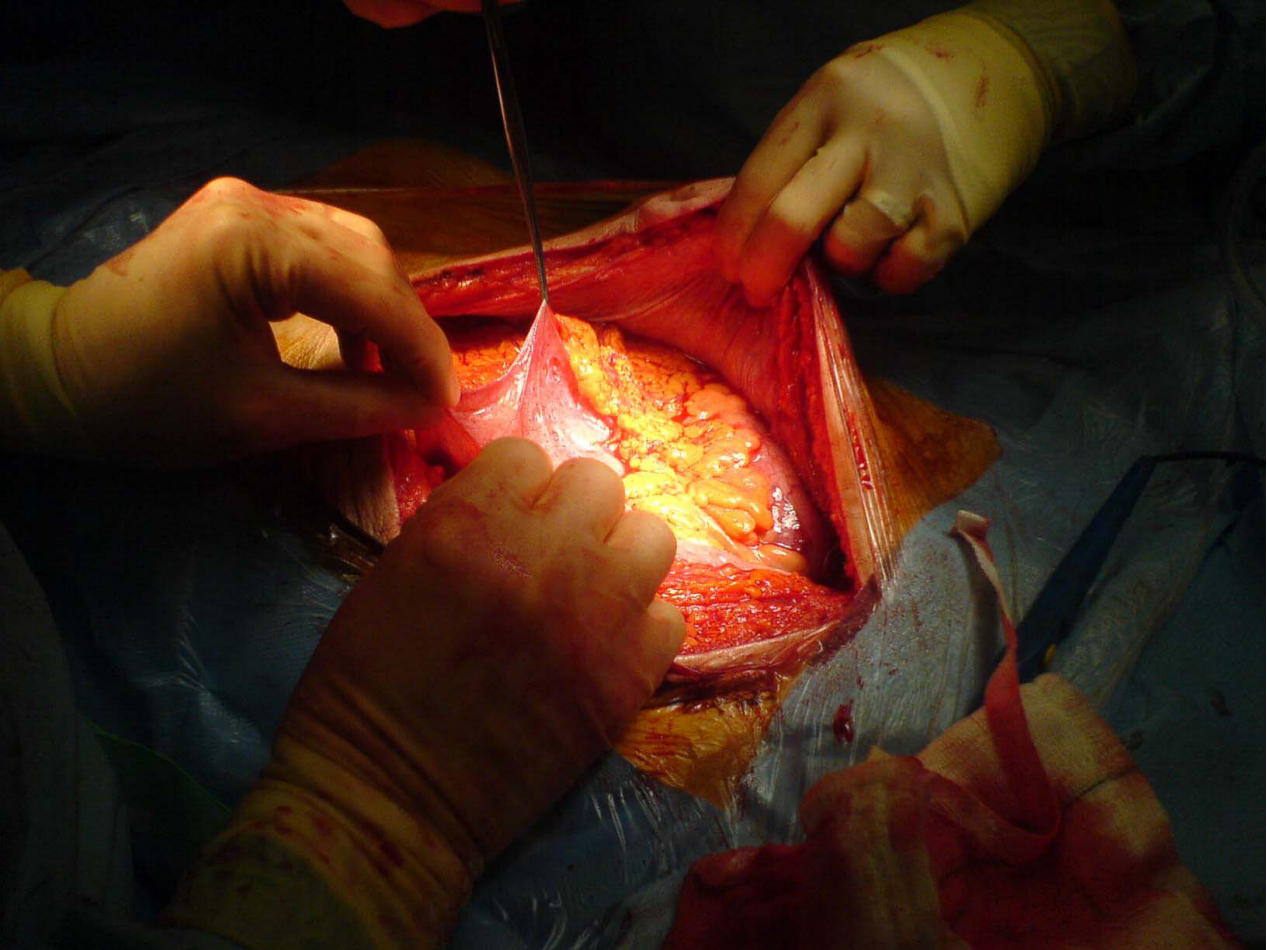
**Peritoneal encaspulation of the small bowel observed after opening the abdomen**.

**Figure 2:**
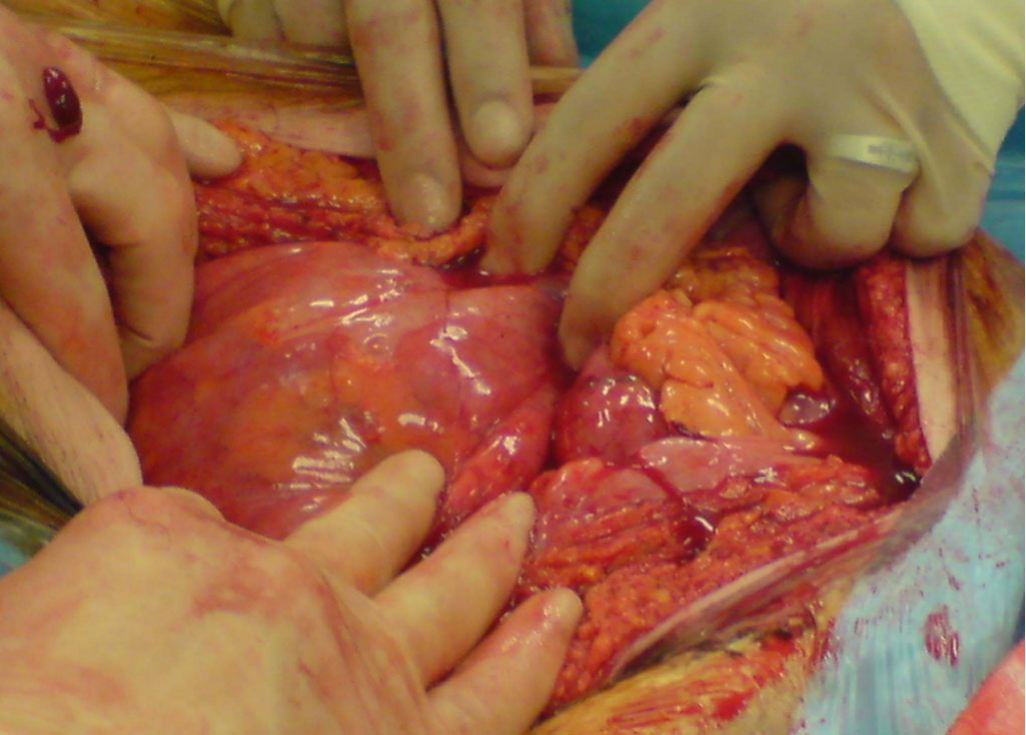
**Small bowel loops visible through the sheet of the peritoneal encapsulation**.

**Figure 3:**
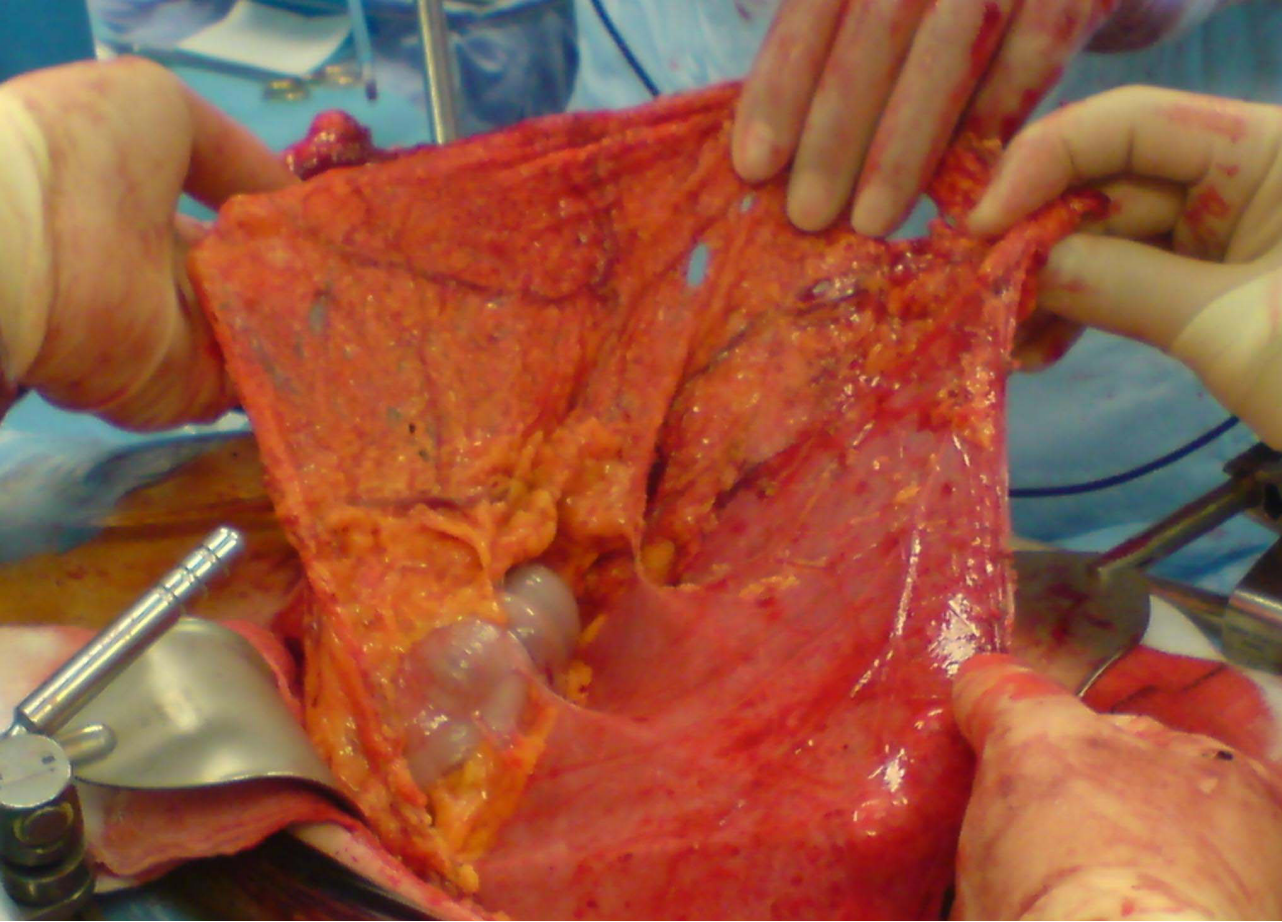
**Superior attachment of PE to the inferior surface of the transverse colon with greater omentum.above, inferior and lateral attachments have been divided**.

**Figure 4:**
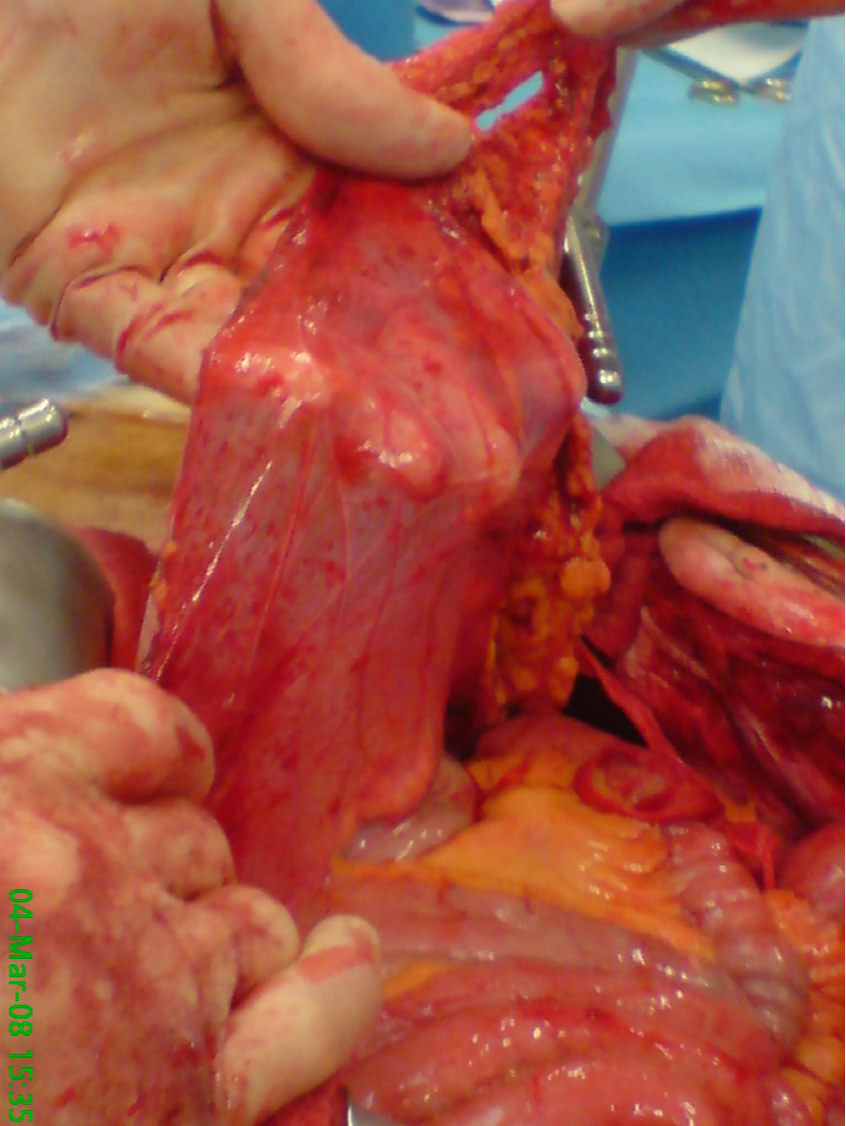
**Membrane of peritoneal encapsulation is being held up by the operating surgeon, with the small bowel visible inferiorly**.

An 82 year old white male presented to the out patient clinic complaining of left iliac fossa pain and was diagnosed with a descending colon cancer. He had never undergone abdominal surgery previously. The tumour was thought to be at risk of obstructing the colon and a potentially curative surgery was planned. During operation and after entering the peritoneal cavity the small bowel was entirely covered with a thin membrane that had the appearance of an accessory peritoneum through which the small bowels were visible (see figure 1, figure 2, figure 3 and figure 4). Excision of the membrane released the small bowels. There was no associated small bowel pathology or adhesions. A palliative Hartmann's procedure was performed as peritoneal metastases were encountered during surgery. The patient made a good recovery post operatively and was discharged from hospital after five days.

## Discussion

Peritoneal encapsulation is a very rare condition characterised by what looks to be an accessory peritoneal membrane covering all or part of the small bowels [1]. This is attached to the ascending and descending colon laterally, the transverse colon superiorly and the posterior surface of the parietal peritoneum inferiorly. It is believed to be caused by mal-rotation of the bowel during the 12^th ^week of gestation [1], this causes the formation of an accessory sac from the peritoneum covering the umbilicus. The membrane may cover the entire or part of the small bowel from the duodeno-jejunal junction down to the ileo-colic junction [2].

The two commonest clinical presentations are: acute small bowel obstruction or incidental diagnosis during laparotomy for another condition [4], however, many cases are incidental findings at autopsy. Some patients may have episodes of intermittent colicky abdominal pain or episodes or sub-acute small bowel obstruction, prior to a definitive diagnosis,

Diagnosis of PE pre-operatively may be impossible because plain abdominal x-ray may be normal or only show dilated small bowel loop and CT scan findings can be very non-specific [3]. Membrane division during surgery is curative (assuming there is no ischaemic bowel) [2]. On literature search no re-operation was reported on PE after dividing the encasing membrane.

Knowledge of this congenital anomaly is of potential use to the abdominal surgeon, as its presence is not reported in standard anatomical descriptions. Its presence is a source of potential confusion, particularly in laparoscopic surgery after establishing pneumo peritoneum. The use of laparoscopy to carry out abdominal surgery is increasing, knowledge of this anatomical anomaly would allow a surgeon, who recognised the membrane as PE, to divide the membrane laparoscopically without recourse to conversion to open laparotomy.

## Consent

Written informed consent was obtained from the patient for publication of this case report and accompanying images. A copy of the written consent is available for review by the Editor-in-Chief of this journal.

## Competing interests

The authors declare that they have no competing interests.

## Authors' contributions

OA prepared the manuscript. ME carried out the literature search and edited the manuscript. JS conceived the idea to write the case report and edited the manuscript. All authors read and approved the manuscript.
